# Morphologically tagged lexical dataset of Khorezm Dialect and Standard Uzbek Pairs

**DOI:** 10.1016/j.dib.2026.113221

**Published:** 2026-09-03

**Authors:** Maksud Sharipov, Lola Kurbanova, Ruzikajon Kurbonova, Elmurod Kuriyozov

**Affiliations:** Urgench State University named after Abu Rayhan Biruni, Khamid Alimdjan 14, 220100, Urgench City, Uzbekistan

**Keywords:** Khorezm dialect, Uzbek language, Machine translation, Parallel corpus, Turkic languages, NLP

## Abstract

In this article, we present a morphologically annotated lexical dataset designed to support the translation of texts between the Khorezm dialect of Uzbek and standard Uzbek. The database consists of 1445 Khorezm dialect–standard Uzbek word pairs. For each entry, the dialect form, its corresponding standard form, and part of speech are provided, along with a morphological structure segmented into affixes according to such grammatical categories as number (singular/plural), possessive, and person/number properties. During the tagging process, the grammatical system of Uzbek and the specific inflectional properties of the Khorezm dialect were taken into account, resulting in a clear and machine-processable layer of correspondences between the dialect and the standard language. The resulting lexical resource is intended to serve as additional input data for AI-based translation and normalization models, as well as in applications for morphological analysis, spelling and grammar checking, and educational tools for teaching the Khorezm dialect. This dataset constitutes the first systematic lexical-morphological resource for corpus-based research on the Khorezm dialect and lays the groundwork for studies on machine translation and automatic alignment between dialects and the standard language in low-resource Turkic varieties.

Specifications Table

**Table:** 

Subject	Computer Science, Linguistics;
Specific subject area	Natural language processing, Computational Linguistics, Corpus Linguistics;
Type of data	Morphologically annotated lexical data (in tabular form)
Data collection	The dataset was constructed on the basis of an existing Khorezm dialect–standard Uzbek dictionary [1]. First, 1445 word pairs were selected from this dictionary in which the dialect form and its corresponding variant in standard Uzbek are clearly specified. During selection, priority was given to the balance of various parts of speech words (noun, verb, adjective, adverb, etc.) and to items that clearly reflect the morphological and phonetic differences characteristic of the Khorezm dialect. The dictionary entries were first digitized (converted into a digital format), normalized according to a unified orthographic and writing standard, and stored in UTF-8 encoding. After that, for each word pair, the part of speech (POS) was assigned, and the stem of the word as well as plural, possessive, and person–number suffixes were segmented as separate units. During the data collection and verification process, manual checking was carried out by experts with strong knowledge of both the Khorezm dialect and standard Uzbek. Duplicate or ambiguous pairs were removed. The final table includes only those dialect–standard word pairs that are semantically compatible and have a clearly defined morphological structure.
Data source location	Country: Uzbekistan Region: Khorezm province (dialect material mainly from Khorezm region and adjacent areas) Languages: Khorezm dialect of Uzbek and standard Uzbek, also the English translation of the words present in the dataset The dataset is built on lexical items extracted from a Khorezm dialect–standard Uzbek dictionary, and the processes of digitization, normalization, and morphological annotation were carried out in Uzbekistan.
Data accessibility	Repository name: Mendeley Data Dataset title: A Morphologically Annotated Lexical Dataset of Khorezm Dialect–Standard Uzbek Word Correspondences Data identification number: 10.17632/6vyt9krx4k.3 Direct URL to the data: https://data.mendeley.com/datasets/6vyt9krx4k/3 Regarding data reuse, while a portion of the baseline vocabulary was sourced from a patented electronic dictionary, the copyright holders have granted explicit permission for this derived, morphologically enriched dataset to be publicly distributed. The dataset is released under the CC BY 4.0 license, permitting future researchers to freely reuse, modify, and integrate the data into NLP pipelines.
Related research article	None

## 1 Background

Dialect–to–standard language translation in morphologically rich languages presents challenges that go beyond simple lexical substitution. Dialectal word forms often differ from their standard counterparts through affix variation, phonological alternation, and non-standard morphological patterns. As shown in computational studies of rich inflectional systems, accurate morphological analysis and disambiguation are essential for resolving form–meaning ambiguity and ensuring reliable downstream processing [2].

Computational morphology has traditionally relied on rule-based finite-state models, which provide interpretable and linguistically grounded analyses [3], as well as data-driven neural approaches that learn morphological structure from annotated examples [4]. Both paradigms depend heavily on high-quality morphologically tagged lexical resources. For under-resourced languages and dialects, however, such datasets remain limited, making corpus construction and manual annotation a critical step [5,6].

Dialectal variation has been extensively studied in various low-resource contexts, where supervised and semi-supervised methods have been applied to map dialect forms to standard varieties [7]. Recent work has also demonstrated that morphological and semantic representations, including word embeddings, can effectively model dialectal differences when supported by structured linguistic annotation [8]. Standardized annotation frameworks such as Universal Dependencies further facilitate cross-lingual and cross-dialectal consistency in morphological tagging [9].

Within this context, the present dataset addresses the lack of morphologically annotated lexical resources for the Khorezm dialect of Uzbek. It provides dialectal word forms aligned with standard Uzbek equivalents and enriched with lemma and morphological feature annotations. The dataset is intended to support AI-based translation, normalization, and morphological analysis models, and to serve as a reusable resource for research on dialect–standard mapping in Turkic languages.

The Khorezm dialect is one of the historically and regionally important varieties of Uzbek and differs significantly from standard Uzbek in its phonetic, lexical, and morphological properties [10,11]. Despite its wide use in everyday communication and in oral folk literature, digital and corpus-based resources for the Khorezm dialect are very limited. As a result, existing Uzbek NLP systems (tokenization, lemmatization, morphological analysis, and translation modules) make many errors when processing units belonging to this dialect [12].

To effectively train AI-based text normalization and machine translation models, a clear, structured, and morphologically rich mapping between the dialect and the standard language is required. For this purpose, a morphologically annotated lexical dataset was created, consisting of 1445 Khorezm dialect–standard Uzbek word pairs, segmented by part of speech and grammatical categories (singular/plural, possessive, and person–number suffixes). This resource, while not many enough to train a neural machine translation model, provides at least a basic lexical layer to support automatic alignment between the dialect and the standard language and is intended to systematically advance both language technologies and linguistic research for the Khorezm dialect.

## 2 Value of the Data

The following are the values of the presented data:

• This dataset is, as per the time of publishing, the first systematic, morphologically annotated lexical resource between the Khorezm dialect and standard Uzbek, filling an important gap in NLP research on low-resource Turkic dialects.
• The explicit alignment between dialect forms and their equivalents in standard Uzbek, together with detailed encoding of inflectional properties such as part of speech and morphological properties, is highly useful for developing and evaluating dialect–standard normalization and supporting rule-based machine translation prototyping, alignment scaffolding, or evaluation.
• Researchers can reuse this lexicon as a gold-standard resource for training and testing morphological analyzers, taggers, and lemmatizers specifically adapted to Uzbek and its regional varieties.
• The dataset can also be applied in educational and pedagogical contexts, for example: digital tools for learning the Khorezm dialect, teaching dialect speakers standard Uzbek, and as practical material in linguistics courses to illustrate morphological differences.
• This resource can serve as a basis for comparative and typological studies of dialect differences, affixation systems, and grammatical categories within the family of Turkic languages, and can support comparative research across different languages and dialects.

## 3 Data Description

Within this study, a dataset was constructed that contains the dialectal forms of words in the Khorezm variety, which belongs to the Oghuz branch of the Uzbek language. This dataset is intended to be used as an input resource in the rule-oriented algorithm described in the following sections.

The dataset is stored as a comma-separated-values (.csv) file that presents words in the Khorezm (Oghuz) dialect and their corresponding forms in standard Uzbek with morphological annotation [13]. Each row contains information about a single lexical unit, and the table is structured with the following columns:

• ***ID*** – sequential index (running index), a unique number for each record.
• ***Khorezm: Word in the Khorezm dialect (base form)*** – the basic (uninflected) form of the word in the Khorezm dialect.
• ***Uzbek: Standard Uzbek form (base form)*** – the corresponding normative form of the same lexical unit in standard Uzbek. This is the one-to-one mapping of the Khorezmian base form.
• ***English***– translation of the base word in English;
• ***POS*** – the grammatical category of the word (e.g., noun, verb, adjective, adverb).
• ***Khorezm-inf: Inflected Khorezm form*** – the form of the word in the Khorezm dialect as it appears in real texts, together with grammatical suffixes (plural, possessive, person–number, case, etc.). While there are many inflected variants of the base form, this column holds only one example of it, usually the most occurring one among the examined corpus.
• ***Uzbek-inf: Inflected standard form*** – the inflected, contextual form of the same unit in standard Uzbek (where available).
• ***Number*** – grammatical category indicating whether the word is in the singular or plural.
• ***Possessive / Person–Number***– information on possessive suffixes and person–number category (e.g., 1st person singular, 3rd person plural, etc.).
• ***Tense***– tense category suffixes for verbs (e.g., present–future, past, etc.) and, where relevant, other temporal/aspectual information for other parts of speech.
• ***Case***– case category suffixes for nouns and substantivized forms (nominative, accusative, dative, ablative, etc.).

Presenting both base (stem) forms and inflected forms in the same row in a tabular format makes it possible to observe lexical and morphological correspondences between the Khorezm dialect and standard Uzbek simultaneously, and to generate explicit input features for both rule-oriented and neural models [14]. While a single base form in the Khorezm dialect can naturally produce multiple inflected variants in running text depending on the syntactic context, this dataset intentionally selects the single most frequently occurring inflected variant observed within the analyzed text corpus for each entry. The inflected forms in standard Uzbek were then generated as direct structural and contextual equivalents to the dialectal inflected forms. This approach provides a clear, end-to-end mapping of morphological transformations while maintaining a structured, one-to-one row alignment between the base and inflected pairs.

To ensure standardization and usability in modern NLP pipelines, the applied Part-of-Speech (POS) tag set and morphological categories were designed to align closely with the Universal Dependencies (UD) framework conventions where applicable to the Turkic language family. The file is stored in UTF-8 encoding and, in line with the Data in Brief format, is ready for direct use in the AI-based translation and normalization systems described in the subsequent sections. However, the MOD (Modal verb) tag was retained as a deliberate local deviation from standard UD (which typically maps modals to AUX or VERB) in order to more accurately capture the specific syntactic behavior of these lexical units in our data.

A detailed description of the morphological analysis of the constructed morphological lexicon is presented in Table 1.

**Table 1:** Detailed description of the tags used in the morphological analysis of the constructed morphological lexicon.

POS tag	Description	Count	Percentage (%)
NOUN	Noun	545	37.72**%**
ADJ	Adjective	244	16.89**%**
PROPN	Proper noun	120	8.3**%**
VERB	Verb	180	12.46**%**
ADV	Adverb	113	7.82**%**
PRON	Pronoun	122	8.44**%**
NUM	Numeral	119	8.24**%**
MOD	Modal verb	2	0.14%
**Total**		**1445**	**100%**

The manual annotation process was conducted by the project's NLP and AI team, with the direct assistance of 12 master's students specializing in Computational Linguistics, supervised by two professors who are experts in the field. Prior to the task, all student annotators underwent a training session focused on standardized guidelines for extracting base forms and uniformly assigning morphological affixes based on standard Uzbek grammatical rules. The second largest portion of the data—around 20%—belongs to the second stage, in which web news texts and literary works available in both varieties were aligned at the sentence level using Hunalign (v1.1), an automatic sentence-alignment tool operating with a baseline Khorezm–Uzbek bilingual dictionary scaffolding. For this text-derived portion, the automatic sentence-level parallel pairs were subsequently reviewed and verified manually by the annotators to ensure exact alignment. From these aligned sentences, standalone, reliable word-level Khorezm-Standard Uzbek lexical pairs were extracted by the annotators. Before merging these newly extracted pairs with the primary dictionary data, a final manual quality check was performed to filter out context-dependent idioms and ensure structural consistency across the unified dataset. Finally, the smallest share (<1%, which is part of the second stage) is taken from an already existing source: Komil Avaz’s book “Xorazm so‘zlarining qisqacha izohli lug‘ati” (“A Short Explanatory Dictionary of Khorezm Words”).

To ensure data reliability, an Inter-Annotator Agreement (IAA) check was performed on a random sample of 150 entries (∼10% of the dataset) (12 master student annotators, plus two experts). Because the evaluation involved multiple independent annotators from the team, Fleiss' Kappa was computed across the core annotation dimensions (including Part-of-Speech tagging and suffix segmentation boundaries), yielding a strong agreement rate of 0.84. Any minor disagreements regarding morphological segmentation or tag mapping during the broader annotation phase were resolved through collaborative discussion and a final consensus verification overseen by the senior researchers.

In the final dataset, there are 1445 words balanced across all morphological varieties, with each Khorezm dialect word and its translation in standard Uzbek placed side by side in each row. As shown in Table 1, morphological analysis reveals that nouns constitute the highest proportion of entries in our lexicon. This suggests that a substantial part of the differences between the Khorezm dialect and the standard language is concentrated in the nominal domain. Whether this pattern is specific only to our limited lexicon or reflects a more global tendency across the entire language system remains an open question for future research. The example snippet of the dataset is shown in Table 2.

**Table 2:** Example snippet from the dataset with original column heads and text samples.

Xorazmcha (Khorezmian)	Adabiycha (Uzbek)	English	POS	Xorazm- Inf (Khorezmian inflectional)	Adabiy-Inf (Uzbek inflectional)	Son (Number)	Egalik/ Shaxs-son (Posessive/ Person-Num)	Zamon (Tense)	Kelishik (Case)
Axtiq	Nevara	Grandson	NOUN	Axtiqlaringiza	Nevaralaringizga	ko`plik	ingiz		ga
Adik	Etik	Boot	NOUN	Adikimni	Etikimni	birlik	im		ni
Al	Qo‘l	Hand	NOUN	Allarimni	Qo‘llarimni	ko`plik	im		ni
Arik	O‘rik	Apricot	NOUN	Ariklarimizdan	O‘riklarimizdan	ko`plik	imiz		dan
Akkal	Olib kel	Bring	VERB	Akkaldik	Olib keldik	ko`plik	k	di	
Ana	Buvi	Grandmother	NOUN	Anamni	Buvimning	birlik	m		ning
Apka	Opa	Elder sister	NOUN	Apkamnan	Opamdan	birlik	m		dan
Aka	Dada	Father	NOUN	Akangni	Dadangni	birlik	ng		ni
Ajina	Jin	Jin	NOUN	Ajinangni	Jiningni	birlik	ing		ni
Aldi	Oldi	Front	ADV	Aldimda	Oldimda	birlik	m		da
Aldin	Oldin	Before	ADV	Aldinnan	Oldindan	birlik			dan

Note that in the Khorezm dialect, the genitive and accusative cases often merge phonetically into the single suffix *-ni*. Therefore, dialectal forms with *-ni* are correctly mapped to standard forms with *-ning* where the syntactic context demands a genitive. This case can be seen in Table 2, in the example Khorezmian lemma “Ana”(Grandmother).

## 4 Experimental Design, Materials and Methods

Before turning to the description of the data collection process, we first outline the linguistic properties of standard Uzbek and the Khorezm dialect, which are the focus of this study, highlighting their common features and main differences.

**The Uzbek language.** Uzbek is a Turkic language belonging to the Karluk branch of the wider Altaic language family. It is spoken primarily in Uzbekistan and also has smaller speaker communities in neighboring Central Asian countries. With >33 million speakers worldwide, Uzbek is one of the most widely used Turkic languages. One of its key characteristics is agglutination, i.e., words are formed by attaching affixes to base morphemes, resulting in complex word structures [15].

**The Khorezm dialect.** The Khorezm dialect is one of the unique regional varieties of Uzbek. It belongs primarily to the Oghuz branch of the Turkic languages, though it historically incorporates minor Karluk and Kipchak elements. It is spoken mainly in the Khorezm region and adjacent areas, and also appears as a minority language in some border districts of Turkmenistan. The fact that nearly two million people speak the Khorezm dialect makes it one of the important dialects of Uzbek. Linguists note that while the Khorezm dialect preserves general Turkic features in its phonetics, lexicon, and grammatical structures, it also contains distinctive elements that set it apart from standard Uzbek.

The lexical similarities and differences between the Khorezm dialect and standard Uzbek are shaped by a range of linguistic factors. Historically, while standard Uzbek developed on a Karluk foundation, the Khorezm dialect developed with a strong Oghuz base. Although the dialect has absorbed some Karluk and Kipchak influences over time through geographical proximity and historical contact, its fundamental Oghuz roots explain its distinct phonetic, morphological, and semantic properties compared to the standard language. Some lexical items in the dialect belong to the older Turkic layer and are either infrequently used in standard Uzbek or expressed through synonyms there [16].

Phonetic features such as vowel shifts, the preservation of diphthongs, and the softening or strengthening of certain consonants give the dialect’s vocabulary a distinct sound pattern. From a morphological perspective, the Khorezm dialect displays specific variants of suffixes, distinctive verb forms, and certain differences in person–number markers.

The lexical layer includes not only words of Turkic origin, but also Arabic, Persian, and Russian elements that entered the language through historical contact. However, many words in the Khorezm dialect differ from the standard language in form, meaning, or usage. For this reason, when compiling a dialect dictionary, the phonetic shape, morphological structure, semantic field, and equivalents in standard Uzbek must be carefully analyzed.

A Khorezm dialect–standard Uzbek dictionary is an important resource for documenting differences between the two varieties on a scientific basis, preserving regional linguistic features, and transmitting them to future generations. Such dictionaries are valuable not only for dialectological research but also for practical fields such as computational linguistics, machine translation, and text normalization.

The morphological lexicon presented in this study is the result of combining three main components, and therefore, the overall process is classified into three stages. Each stage is described in detail in the following sections. All co-authors of the article actively participated in the construction of the morphological lexicon. The linguistic experts among the authors were responsible for checking the spelling of the dialect words, identifying their official equivalents, and carrying out the morphological analysis.

At the initial stage of creating the dataset, the second and third columns were compiled; these contain, respectively, the Khorezm dialect words in Latin script and their standard Uzbek equivalents in Latin script. The initial dictionary-based extraction stage accounts for approximately 80% of the dataset. At the next stage, Uzbek language specialists assigned each item to a part of speech and added the corresponding tags in the fourth column.

**Fig. 1:**
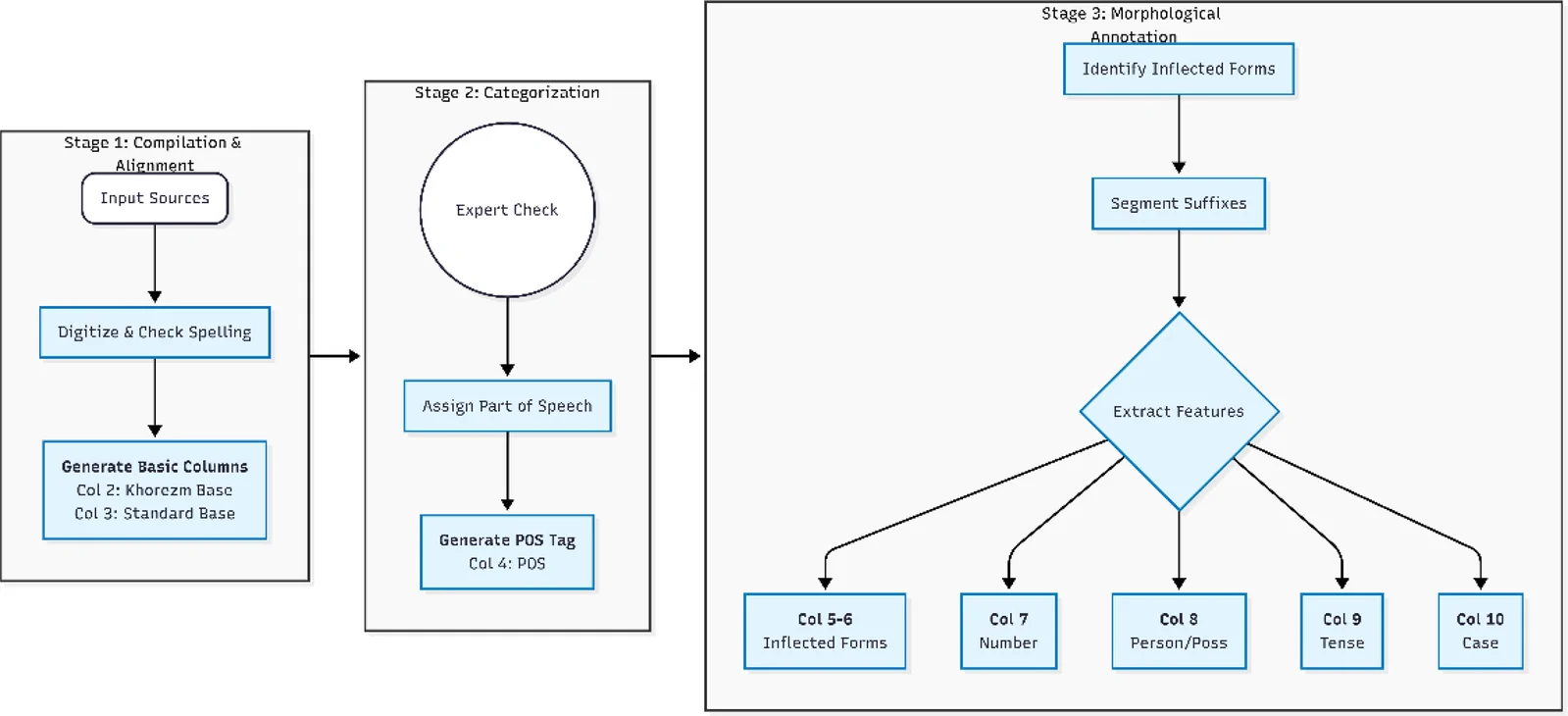
Algorithmic process steps of the dataset creation.

Finally, the fifth and sixth columns contain the inflected forms of the dialect words and their official equivalents in standard Uzbek: the fifth column lists the words in the Khorezm dialect in Latin script, and the sixth column provides their counterparts in standard Uzbek in Latin script. In addition, the seventh column indicates whether each word is in the singular or plural. In the eighth column, person–number suffixes are identified and recorded separately for each word. The ninth column specifies the tense categories, and in the tenth (final) column, case suffixes are determined for each word and entered by language specialists. All the steps involved in the dataset creation process are presented in Fig. 1.

## Limitations

Despite its usefulness, the dataset has certain inherent limitations. One of the major drawbacks of this dataset is the fact that the collected lexical material reflects only a subset of the Khorezm dialect, meaning that the other regional or speaker-specific variants may be missing. This can influence how well AI models generalize to unseen dialectal forms.

Another limitation could be that the dataset is mainly lexical in nature, with limited contextual or sentence-level information. This restricts its ability to fully support semantic disambiguation and pragmatic interpretation in advanced neural translation models.

In addition, lexical entries are unevenly distributed across morphological categories, with greater emphasis on frequently used nouns and verbs than on function words or expressive dialectal units. The lack of phonological or speech-related features also limits direct use in spoken language applications.

The existence of these limitations mentioned above does not lower the value of the dataset in any case and future extensions will focus on broader dialect coverage, richer contextual annotation, and the inclusion of phonetic or multimodal data to improve robustness and applicability.

## Ethics Statement

During the creation of the dataset, no medical, biometric, or sensitive personal data were collected or analyzed. Where any language examples originated from human contributors or community sources, participation was voluntary and informed, and content was reviewed to avoid inclusion of personally identifiable information. Any identifiers (names, phone numbers, addresses, or other traceable details) were removed during data cleaning. The dataset is shared in aggregated lexical form and is intended for scientific research and language technology development.

The authors recognize that dialect resources can be culturally sensitive. The dataset is released to support language preservation and equitable NLP development, and it should not be used to stereotype, profile, or harm individuals or communities. Users are expected to comply with applicable data protection regulations and to provide appropriate attribution when using the dataset.

## Credit Author Statement

The contributions of this work are distributed among the authors as follows:

• **Maksud Sharipov:** Conceptualization, Methodology, Data Curation.
• **Lola Kurbanova:** Software, Validation, Dataset publication, Resources.
• **Ruzikajon Kurbonova:** Investigation, Writing - Original Draft, Visualization.
• **Elmurod Kuriyozov:** Writing - Manuscript preparation, Proofreading, Formal analysis.

## Data Availability

Mendeley DataA Morphologically Annotated Lexical Dataset of Khorezm Dialect–Standard Uzbek Word Correspondences (Original data).
